# Correlation between Rotator Cuff Tears and Systemic Atherosclerotic Disease

**DOI:** 10.1155/2011/128353

**Published:** 2011-10-19

**Authors:** Andrea Donovan, Mark Schweitzer, Jenny Bencardino, Catherine Petchprapa, Jodi Cohen, Gina Ciavarra

**Affiliations:** ^1^Department of Radiology, Sunnybrook Health Sciences Centre, Room AG 278, 2075 Bayview Avenue, Toronto, ON, Canada M4N 3M5; ^2^Department of Diagnostic Imaging, The Ottawa Hospital—General Campus, 501 Smyth Road, Ottawa, ON, Canada K1H 8L6; ^3^Department of Radiology, NYU Hospital for Joint Diseases, 6th Floor, 301 East 17th Street, New York, NY 10003, USA

## Abstract

The purpose of this study was to investigate the association of aortic arch calcification, a surrogate marker of atherosclerosis, with rotator cuff tendinosis and tears given the hypothesis that decreased tendon vascularity is a contributing factor in the etiology of tendon degeneration. A retrospective review was performed to identify patients ages 50 to 90 years who had a shoulder MRI and a chest radiograph performed within 6 months of each other. Chest radiographs and shoulder MRIs from 120 patients were reviewed by two sets of observers blinded to the others' conclusions. Rotator cuff disease was classified as tendinosis, partial thickness tear, and full thickness tear. The presence or absence of aortic arch calcification was graded and compared with the MRI appearance of the rotator cuff. The tendon tear grading was positively correlated with patient age. However, the tendon tear grading on MRI was not significantly correlated with the aorta calcification scores on chest radiographs. Furthermore, there was no significant correlation between aorta calcification severity and tendon tear grading. In conclusion, rotator cuff tears did not significantly correlate with aortic calcification severity. This suggests that tendon ischemia may not be associated with the degree of macrovascular disease.

## 1. Introduction

An understanding of the pathogenesis of rotator cuff disease has advanced greatly over the last 40 years since Neer first postulated that the rotator cuff tears result from acromion undersurface abrasion [1]. Several intrinsic and extrinsic factors have been implicated in the pathogenesis of rotator cuff tears, but the relative importance of each component remains disputed. 

The original extrinsic osseous mechanical impingement theory has recently been challenged following demonstration that acromial shape [2, 3] and subacromial spurs [4] are not the primary cause of shoulder impingement syndrome or rotator cuff tears. There is evidence that intrinsic tendon degeneration, likely related to decreased tendon vascularity and associated ischemia, leads to rotator cuff tears [5–7]. Furthermore, increasing patient age may also be a risk factor for rotator cuff degeneration, with the vast majority of tears occurring in patients over 50 years old [3, 8, 9]. However, systemic atherosclerosis has not yet been specifically studied in relation to rotator cuff disease. 

The purpose of this study was to evaluate whether chest radiograph findings of aortic arch calcification, a surrogate marker of atherosclerosis [10], is associated with rotator cuff disorders as identified by shoulder magnetic resonance (MR) imaging.

## 2. Materials and Methods

### 2.1. Patient Population

Institutional review board approval was granted, and informed consent was waived for this retrospective Health Insurance Portability and Accountability Act-compliant study. A radiology database was retrospectively reviewed to identify subjects between ages 50 and 80 years old, with MRI of the shoulder and a chest radiograph performed within 6 months of the shoulder MRI exam. Previous studies have shown that the prevalence of rotator cuff tears increases with age [9], as does the visualization of aortic arch calcifications on chest radiographs [10]. Therefore, the study population comprised subjects between 50 and 80 years old to ensure a sufficient number of study subjects with rotator cuff tears as well as aortic arch calcifications.

The population size was determined based on the sample size calculation, where sample size of 100 subjects would have power of 80% to yield a statistically significant result. This search spanned a 3-year period from January 2004 to February 2007. Additional exclusion criteria included prior rotator cuff surgery, chest radiograph technically inadequate to assess for aortic arch calcification, and incomplete or unavailable imaging studies on our Picture Archiving and Communication System (PACS). 

The study group comprised 120 patients: 67 females and 53 males, mean age 68 years for both genders, (range 50–80 years for females, 51–80 years for males). Medical records were reviewed, and clinical details recorded included patient age, gender, and involved side. MRI studies were evaluated by two musculoskeletal radiologists, with 8 and 13 years of experience in interpretation of shoulder MRI, respectively. The observers were blinded to the others' conclusions as well as to the clinical information. Two additional radiologists evaluated chest radiographs, both with 5 years of experience in interpretation of chest radiographs. Similarly, the chest radiograph observers were blinded to the others' conclusions as well as to the clinical history and shoulder MRI findings.

### 2.2. MRI Technique

The shoulder MRI studies were performed on several different MR units (Siemens, Erlangen, Germany); nearly all patients were performed at 1.5 T. Although the imaging protocols were slightly different, the MR protocol in all patients consisted of two coronal oblique acquisitions (intermediate-weighted and T2-weighted fast-spin echo with fat suppression), two sagittal oblique acquisitions (T1-weighted and intermediate-weighted with fat suppression), and a single axial plane (intermediate-weighted with fat suppression). T1-weighted images were acquired with TR/TE of 400–700/10–20 ms; intermediate-weighted images were obtained with TR/TE (effective) of 2500–3800/34–38 ms; T2-weighted fast spin-echo images were acquired with a TR/TE (effective) of 2000–6000/60–90 ms.

### 2.3. MRI Interpretation

**Figure 1:**
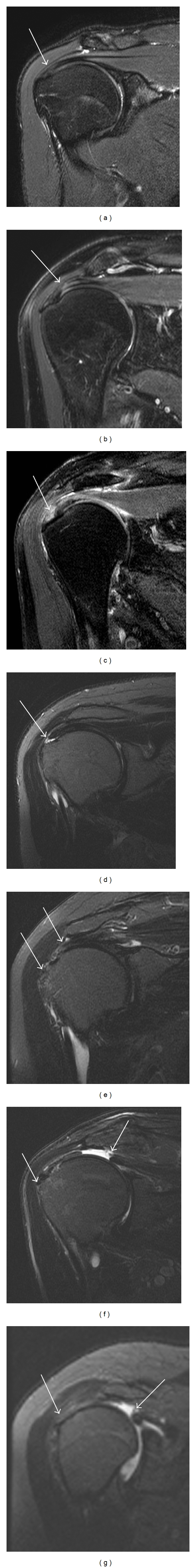
Shoulder MRI grading of rotator cuff pathology. Seven coronal oblique T2-weighted, fat-suppressed MR images frontal illustrate the grading scheme of rotator cuff pathology. Arrows show the supraspinatus tendon. (a) Grade 0: normal rotator cuff tendon; (b) grade 1: tendinosis; (c) grade 2: partial thickness tear; (d) grade 3: small full thickness tear (less than 1 cm); (e) grade 4: medium full thickness tear (between 1 to 3 cm); (f) grade 5: large full thickness tear (greater than 3 cm but less than 5 cm); (g) grade 6: massive rotator cuff tear (measuring greater than 5 cm and/or involving more than two tendons).

MRI appearance of rotator cuff was assessed, and rotator cuff pathology was graded by two musculoskeletal radiologists. The observers graded the studies independently, blinded to initial interpretation and chest radiographic findings, on a 1–6 scale based on previously described classification [11]: grade 0, normal rotator cuff tendons; grade 1, tendinosis; grade 2, partial thickness tear; grade 3, small full thickness tear (less than 1 cm); grade 4, medium full thickness tear (between 1 to 3 cm); grade 5, large full thickness tear (greater than 3 cm but less than 5 cm); grade 6, massive rotator cuff tear (measuring greater than 5 cm and/or involving more than two tendons) (Figure 1).

### 2.4. Chest Radiograph Interpretation

**Figure 2:**
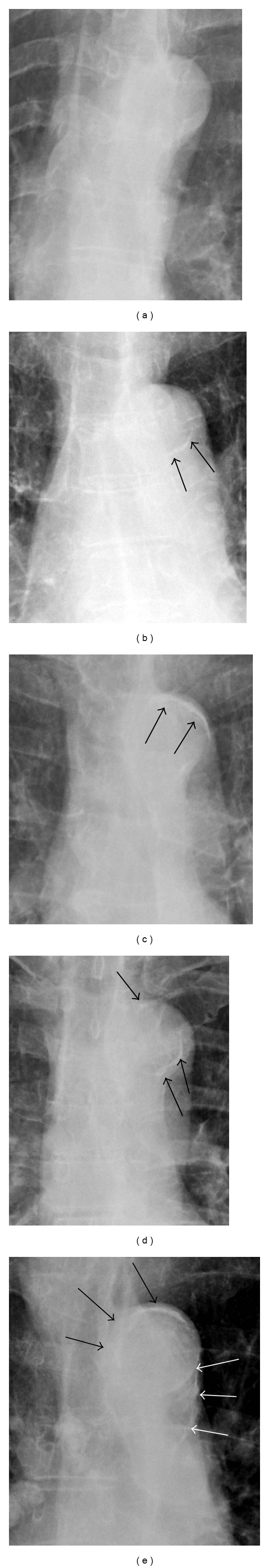
Chest radiographs grading of aortic arch calcification. Five frontal radiographs illustrate the grading scheme of aortic calcification. Arrows show calcifications along the aortic arch. (a) Grade 0: no calcifications visible; (b) grade 1: calcification length between 0-1 cm; (c) grade 2: length between >1-2 cm; (d) grade 3: length between >2-3 cm; and (e) grade 4: length greater than 3 cm in length.

Frontal chest radiographs, including posterior-anterior and portable anterior-posterior examinations, were evaluated independently by two radiologists. Radiographs were assessed for the presence or absence of aortic arch calcification. Aortic calcification (AC) grading on plain chest radiography was performed using a modified scale previously described by Li et al. [12]: grade 0, no calcifications visible; grade 1, calcification length between 0-1 cm; grade 2, length between >1-2 cm; grade 3, length between >2-3 cm; grade 4, length greater than 3 cm in length. In cases where calcifications were discontinuous, total length was obtained by adding the length of separate linear calcific densities along the aortic arch (Figure 2).

### 2.5. Statistical Analysis

Kappa coefficients were used to assess interobserver agreement with respect to the rotator cuff grade on MRI and aortic arch calcification on chest radiographs. Since there were multiple categories, weighted kappa coefficients were calculated. Mixed model analysis of covariance (ANCOVA) was used to evaluate the association of the rotator cuff tendon grading with the aorta calcification grading, while controlling for patient age. The Spearman rank correlation coefficient was used to characterize the strength of the correlation of the rotator cuff grading with patient age and the aorta calcification scores. Statistical computations were performed using SAS version 9.0 (SAS Institute, Cary, NC, USA). Statistical significance was defined as a *P* value less than 0.05.

## 3. Results

### 3.1. Distribution of Rotator Tear Grading

**Figure 3:**
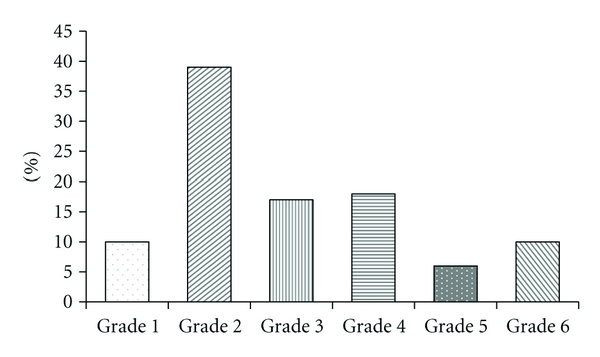
Distribution of rotator tear grading. The bar graph illustrates the distribution of rotator cuff tears. The study group included all rotator cuff tear grades, with the largest proportion of grade 2 tears. There were no normal rotator cuffs included in the study.

The study group included all rotator cuff tear types, with the largest proportion having grade 2—partial tear (*n* = 47; 39%), grade 3—small full thickness tear (*n* = 20, 17%), and grade 4—medium full thickness tear (*n* = 22, 18%), and a smaller proportion having grade 5—large full thickness tear (*n* = 7, 6%) and grade 6—massive tear (*n* = 12, 10%) (Figure 3). There was a smaller proportion of cases with tendinosis, or grade 1 rotator cuff tendons (*n* = 12, 10%), and no cases were scored as being normal, or grade 0, likely related to the patients ages. This distribution represents average numbers of cases in each grade category based on grading performed by both observers.

### 3.2. Distribution of Aortic Arch Calcification Grading

**Figure 4:**
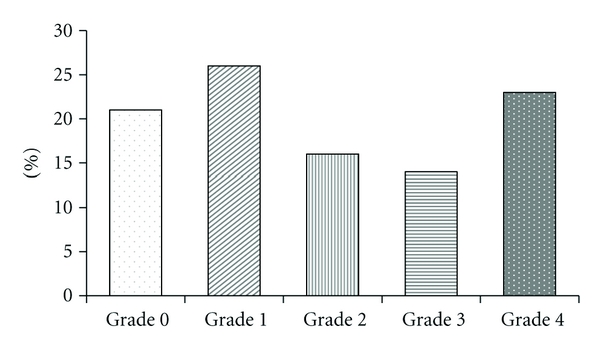
Distribution of aortic calcification grading. The bar graph illustrates the distribution of aortic calcification grading. Aortic arch calcification was distributed relatively equally.

Aortic arch calcification was distributed relatively equally among different grades: grade 0 (*n* = 25, 21%), grade 1 (*n* = 31, 26%), grade 2 (*n* = 20, 16%), grade 3 (*n* = 17, 14%), and grade 4 (*n* = 27, 23%) (Figure 4). Similar to distribution of rotator cuff tears, this distribution represents average numbers of cases in each grade category based on grading performed by both observers.

### 3.3. Association between Rotator Cuff Tear and Patient Age

Tendon tear scores were positively correlated with patient age (correlation: *r* = 0.397; *P* < 0.001).

### 3.4. Association between Rotator Cuff Tear and Patient Gender

According to the mixed model ANCOVA, there was no significant difference between patient genders (*P* = 0.540) in terms of the tendon tear scores from MR.

### 3.5. Association between Rotator Cuff Tear Severity and Grading of Aortic Arch Calcification

 The tendon tear scores were not significantly correlated with mean (over observers) aorta calcification scores (*r* = 0.196, *P* = 0.586). Furthermore, when the aorta calcification scores was analyzed as a classification factor, there were no significant differences among the aorta calcification classes in terms of the tendon tear scores (*P* = 0.163 for the 4-degree-of-freedom composite test for class differences). When aorta calcification was classified as normal (score = 0) versus abnormal (score > 0), the difference between the normal and abnormal groups in terms of tendon severity score was not significant (*P* = 0.171).

### 3.6. Interobserver Agreement

Kappa coefficients were calculated to assess interobserver agreement with respect to rotator cuff grading on MRI and aorta calcification grading on chest radiographs. The weighted kappa coefficient for rotator cuff grading was 0.57 (moderate). The greatest discrepancy was in distinguishing between grade 3 and 4 tears. For example, observer 1 recorded 28 cases as grade 3 and 11 cases as grade 4, whereas observer 2 recorded 11 cases as grade 3 and 28 cases as grade 4. 

The weighted kappa coefficient for chest radiograph evaluation of aortic calcification was 0.71 (substantial) [13].

## 4. Discussion

Rotator cuff pathology is common in the general population [8]. The etiology of rotator cuff disease continues to be debated, but is likely multifactorial. Commonly implicated factors in the development of rotator cuff disease include tendon ischemia [6], extrinsic compression [14], and chronic repetitive microtrauma. However, their relative contribution and the initiating factors are not clear. It is important to understand the causes of rotator cuff tendinopathy to facilitate diagnosis and outcomes, as well as potentially address disorder progression. 

Vascular insufficiency of the supraspinatus tendon and secondary microcirculatory disturbances have been suggested to predispose to rotator cuff tears, shoulder pain, and reduced shoulder function [15]. It is unclear whether tendon ischemia is the cause or the consequence of rotator cuff disease. Prior studies evaluated in vitro and in vivo vascularity of the normal and degenerated rotator cuff [16–18]. However, to our knowledge, this is the first study to evaluate the potential relationship between rotator cuff degeneration and systemic atherosclerosis, as determined by aortic calcifications on chest radiographs. 

Our data support prior studies showing an increased prevalence of rotator cuff disease with patient age [9, 19]. In the current study, there was a statistically significant increase in the tendon score severity with increasing patient age. This age-dependent increase in rotator cuff pathology may imply vascular pathogenesis of intrinsic tendon degeneration, “wear and tear” [20]. Contrast-enhanced ultrasound demonstrated an age-related decrease in the blood supply to the intact, asymptomatic rotator cuff [20].

Risk factors for rotator cuff tears have been studied only recently. A systematic review in 2007 evaluated an association between risk factors of atherosclerosis (diabetes, smoking, and body mass index) and shoulder pain. There was a consistent association with diabetes and shoulder disorders. Additional risk factors for vascular disease such as smoking [21], hypercholesterolemia [22], and increased body mass index [23] are also found to be associated with rotator cuff tendinopathy. 

In this study, we evaluated the relationship between rotator cuff disease and systemic atherosclerotic disease. Aortic calcifications on routine radiographs have been shown to reflect advanced atherosclerotic disease [24]. In addition, aortic calcifications as seen radiographically have been shown to be associated with increased risk of cardiovascular mortality [10] and independently related to coronary disease risk in both men and women [10]. Scoring of aortic calcification on imaging has been used in prior studies to evaluate an association between systemic atherosclerosis and disc disease [25], and systemic atherosclerosis and osteoporosis [26]. Therefore, in this study, we used aortic calcification score on chest radiographs as a surrogate marker of systemic atherosclerosis. We found that aortic arch calcification scores did not correlate with the presence of rotator cuff tear or with tendon score severity. This may suggest that decreased rotator cuff vascularity in diseased tendon is related to microvascular, rather than macrovascular disease (i.e., aortic arch calcification) or perhaps not be related to tendon degeneration at all. 

Historically, tendon degeneration is thought to occur mainly in the areas of poor blood supply. Several cadaveric studies identified a watershed zone near the supraspinatus tendon footprint, the so-called “critical zone” [27–29]. This critical zone is situated at the articular surface as the tendon approaches the greater tuberosity footprint. Conversely, Moseley and Goldie [16] studied the critical zone and found rich vascular anastomoses between osseous and tendinous vessels. Rathburn and Macnab [28] showed that although, in adduction, the supraspinatus tendon vessels were occluded, they were perfused with the arm abducted. In vivo study by Levy with laser Doppler flowmetry did not demonstrate a functional “critical zone of hypoperfusion” in the normal tendon [18]. They attributed the “critical zone” in cadavers to an artifact of the injection technique in cadavers. Most cadaveric studies evaluated vessels >20 *μ*m in diameter whereas laser Doppler flowmetry allows measurements of vessels of 10 *μ*m. These smaller vessels, detected on laser Doppler flowmetry, appear to play a significant role in rotator cuff perfusion. 

 In patients with impingement, however, laser Doppler flowmetry did demonstrate areas of hypoperfusion within the rotator cuff tendon. In patients with rotator cuff tears, both cadaveric and in vivo studies showed an association between rotator cuff tears and decreased tendon tissue vascularity [7, 15]. At arthroscopy, orthogonal polarization microscopy demonstrated a statistically significant decrease in microcirculation adjacent to rotator cuff lesions [15]. Based on the above studies, it is uncertain whether vascularity is the primary cause or an effect of rotator cuff disease. 

Prior studies evaluating tissue vascularity at the site of tear, rather than adjacent to the tear, demonstrated increased vascularity as determined by histology [30] and Doppler flow exams [18]. The discrepancy between hypovascularity around the tear and hypervascularity at the site of the tendon tear has been attributed to neovascularization within tendons. Therapies have been developed to target hypervascularity with the goal to alleviate symptoms. For example, injections of small vessels with sclerosing polidocanol to treat chronic painful shoulder resulted in a significant decrease in visual analogue scale (VAS) score in a study of fifteen patients [31]. 

There are several limitations to this study. First, our study design did not include correlation of shoulder MRI with arthroscopic findings or clinical symptoms. However, the accuracy of MR in diagnosing rotator cuff tears is well established [30] and as we sought to study the true range of cuff disorders, limiting our population to those with surgery would have created selection bias. Second, additional relevant clinical history including smoking, cholesterol levels, body mass index, or history of diabetes was not included. These may represent independent risk factors for the development of rotator cuff disease. Third, the quality of chest radiographs, specifically, penetration, differed, and may have affected the conspicuity of aortic calcifications for any given patient. Lastly, we did not measure and correlate rotator cuff vascularity to aortic arch calcium score or tendon score. In future studies, it would be informative to directly measure tendon vascularity. This could be achieved with contrast ultrasound as previously described by Adler et al. [17]. 

In conclusion, the presence of aortic calcification did not correlate with the presence and severity of rotator cuff tears in this study, suggesting either that vasculopathy is not a risk factor for cuff disease or that microvascular, rather than macrovascular disease, contributes to the pathogenesis of rotator cuff tendinopathy.
